# Predicting Peri-Operative Outcomes in Patients Treated with Percutaneous Thermal Ablation for Small Renal Masses: The SuNS Nephrometry Score

**DOI:** 10.3390/diagnostics13182955

**Published:** 2023-09-15

**Authors:** Gennaro Musi, Stefano Luzzago, Giovanni Mauri, Francesco Alessandro Mistretta, Gianluca Maria Varano, Chiara Vaccaro, Sonia Guzzo, Daniele Maiettini, Ettore Di Trapani, Paolo Della Vigna, Roberto Bianchi, Guido Bonomo, Matteo Ferro, Zhe Tian, Pierre I. Karakiewicz, Ottavio de Cobelli, Franco Orsi, Mattia Luca Piccinelli

**Affiliations:** 1 Department of Urology, IEO European Institute of Oncology, IRCCS, Via Ripamonti 435, 20141 Milan, Italy; gennaro.musi@ieo.it (G.M.); ettore.ditrapani@ieo.it (E.D.T.);; 2 Department of Oncology and Haemato-Oncology, Università degli Studi di Milano, 20122 Milan, Italy; 3 Division of Interventional Radiology, IEO European Institute of Oncology, IRCCS, Via Ripamonti 435, 20141 Milan, Italy; 4 Cancer Prognostics and Health Outcomes Unit, Division of Urology, University of Montréal Health Center, Montréal, QC H2X3E4, Canada

**Keywords:** nephrometry score, percutaneous thermal ablation, SuNS, trifecta

## Abstract

Our objective was to develop a new, simple, and ablation-specific nephrometry score to predict peri-operative outcomes and to compare its predictive accuracy to PADUA and RENAL scores. Overall, 418 patients were treated with percutaneous thermal ablation (microwave and radiofrequency) between 2008 and 2021. The outcome of interest was trifecta status (achieved vs. not achieved): incomplete ablation or Clavien–Dindo ≥ 3 complications or postoperative estimated glomerular filtration rate decrease ≥ 30%. First, we validated the discrimination ability of the PADUA and RENAL scoring systems. Second, we created and internally validated a novel scoring (SuNS) system, according to multivariable logistic regression models. The predictive accuracy of the model was tested in terms of discrimination and calibration. Overall, 89 (21%) patients did not achieve trifecta. PADUA and RENAL scores showed poor ability to predict trifecta status (c-indexes 0.60 [0.53–0.67] and 0.62 [0.55–0.69], respectively). We, therefore, developed the SuNS model (c-index: 0.74 [0.67–0.79]) based on: (1) contact surface area; (2) nearness to renal sinus or urinary collecting system; (3) tumour diameter. Three complexity classes were created: low (3–4 points; 11% of no trifecta) vs. moderate (5–6 points; 30% of no trifecta) vs. high (7–8 points; 65% of no trifecta) complexity. Limitations include the retrospective and single-institution nature of the study. In conclusion, we developed an immediate, simple, and reproducible ablation-specific nephrometry score (SuNS) that outperformed PADUA and RENAL nephrometry scores in predicting peri-operative outcomes. External validation is required before daily practice implementation.

## 1. Introduction

The incidence of kidney cancer is increasing worldwide [1]. Specifically, incidental detection during cross-sectional imaging exams determined higher diagnoses of small renal masses (SRMs) in the last decades [2]. Radical or partial nephrectomy (PN) still represent the first-choice treatment [3], while image-guided percutaneous thermal ablation (PTA) has been proposed as an effective minimally invasive alternative, with reported good oncologic results and low complications rates [4,5,6].

Several scoring systems (nephrometry scores: NSs) exist for stratifying kidney cancer patients into complexity categories predicting complications and oncologic outcomes [7,8,9,10]. These scores are mostly based on easily collectable, preoperative tumour characteristics, not including patients’ clinical parameters, to assess the complexity of the procedure along with short- and mid-term outcome predictions. Moreover, NSs represent the tools to standardize tumour characteristics in clinical practice and research. To date, the PADUA [7] and RENAL [8] NSs, developed on surgically treated cohorts, are the most widely used and established methods to assess the complexity of SRM. However, their application to PTA produced conflicting results. An association between NS and complications [11,12,13], estimated glomerular filtration rate (eGFR [14]) and oncologic outcomes [15,16,17] has been reported by some studies, but not confirmed by others [18,19,20]. A few ablation-specific NSs have been developed but none of them are widely accepted in clinical practice [15,18,19,21,22].

We hypothesized that a new, simple, ablation-specific score (SuNS) might better predict peri-operative outcomes, compared to PADUA and RENAL NSs. To address this hypothesis, we relied on a large (n = 418) contemporary cohort of patients treated at a single institution with PTA for SRMs.

## 2. Materials and Methods

### 2.1. Patient Selection

This retrospective single-institution data analysis was approved by the Institutional Review Board of the European Institute of Oncology.

Overall, 418 patients with SRMs (T1a-b kidney cancer) were treated with PTA between 2008 and 2021 in a tertiary referral center. We focused on patients aged ≥18 years who had undergone either radiofrequency (RF) or microwave (MW) ablation. Age, sex, age-adjusted Charlson comorbidity index (CCI), body mass index, SRM side, location, diameter, T stage, contact surface area (CSA [23,24,25]), nearness to renal sinus, urinary collecting system, ureter, bowel, skin-to-tumour distance, and histology were recorded. Analyses did not include residual or recurrent SRM.

### 2.2. Thermal Ablation Procedure

PTA technique has been previously described [26]. All procedures were performed under general anesthesia, in a dedicated operating room equipped with both ultrasound (US) and computed tomography (CT) scan, with the patient lying in the most favourable position for a direct needle approach. All procedures were performed by a team of two interventional radiologists, with at least one with more than ten years of experience. Adjunct procedures such as hydrodissection or pyeloperfusion were used when appropriate upon team clinical judgment [5]. Procedures were guided by US and CT or by US-CT fusion imaging [27]. Ablation was performed with an RF 3000 system (Boston Scientific, Natick, Massachusetts, MA, USA) and a retrievable hook-umbrella needle or with an MW system (Emiprint, Medtronic, Minneapolis, MN, USA). In cases of suspicious persistence of pathological tissue, device repositioning and retreatment were performed in the same manner as previously described. A contrast-enhanced CT (CECT) scan was performed to evaluate treatment results, ablation completeness and possible complications at 24 h from PTA. Blood tests, including complete blood count, creatinine and eGFR, were performed 24 h and 48 h after PTA. Patients were then followed according to standard protocols with a CT and a clinical visit at 6 weeks, 3, 6, 12, 18 and 24 months and yearly thereafter.

### 2.3. Variables of Interest

CSA was originally described by Leslie et al. in a PN cohort [23]. It can be easily computed using the formula 2πrd (r: radius, d: depth of invasion 24). We modified the CSA cut-off (previously defined as <20 vs. ≥20 cm^2^) to <10 vs. ≥10 cm^2^ accounting for a smaller median size of SRMs in a PTA cohort. RENAL and PADUA NS and derived complexity classes were assigned to all patients as previously described [7,8]. The outcome of interest for both validation and development was trifecta status (achieved vs. not achieved). Trifecta not achieved outcome was defined as follows: partial SRM ablation detected with CECT after 24 h from procedure OR postoperative complication defined as Clavien–Dindo ≥ 3 [28] OR postoperative eGFR decrease ≥ 30% [29].

### 2.4. Statistical Analysis

First, we compared the demographic, radiologic, pathologic and outcome differences, stratifying the cohort according to trifecta status. Second, multivariable logistic regression models predicting trifecta status according to PADUA and RENAL scoring systems [7,8] were fitted and the discrimination ability of both scores was estimated using Harrell’s c-index. Third, separate univariable logistic regression models tested the association between available SRM features and trifecta status. Fourth, statistically significant predictors of trifecta status were used to fit a multivariable logistic regression model (SuNS). Fifth, the predictive accuracy of the model was evaluated with respect to discrimination (i.e., the ability of the model to classify a patient achieving from one not achieving trifecta) using Harrell’s c-index and calibration (i.e., the agreement between the outcome frequencies observed in the data and the predicted probabilities of the model) depicted with the loess plot. Additionally, 2000 bootstrap resamples were performed to PADUA, RENAL and SuNS c-indexes. Finally, a sensitivity analysis was conducted to evaluate discrepancies in the SuNS model ability to predict trifecta status according to the ablation technique. All statistical tests were two-sided, with the level of significance set at *p* < 0.05 and were performed with R Software Environment for Statistical Computing and Graphics (R version 4.1.3, R Foundation for Statical Computing, Vienna, Austria).

## 3. Results

### 3.1. Descriptive Characteristics

Of 418 ablation-treated SRM patients, 89 (21%) did not achieve trifecta (Table 1).

Patients that did not achieve trifecta were older (73 vs. 67 years; *p* < 0.001), less frequently male (57 vs. 69%; *p* = 0.04) and had higher CCI (6 vs. 5; *p* = 0.01), compared to their trifecta-achieving counterparts. SRMs in patients not achieving trifecta were bigger (54 vs. 25% >3 cm; *p* < 0.001), had a more extended CSA (73 vs. 41% ≥ 10 cm^2^; *p* < 0.001) and were closer both to renal sinus/urinary collecting system (43 vs. 15% ≤ 4 mm; *p* < 0.001) and ureter (48 vs. 55 mm; *p* = 0.004).

**Figure 1:**
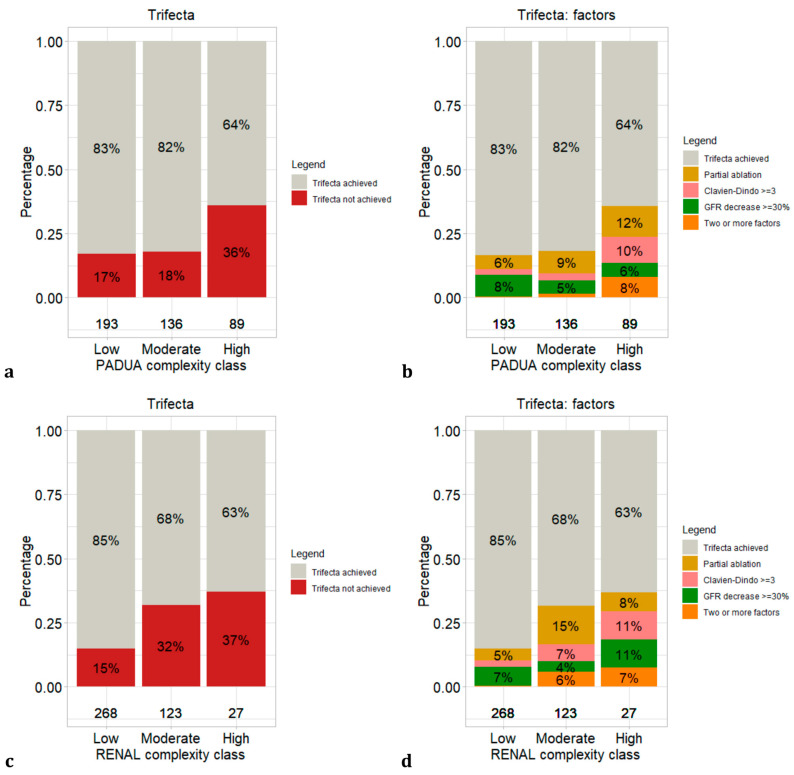
Stacked bar plots depicting trifecta not achieved and factors determining trifecta status distribution according to complexity classes: (**a**) trifecta status according to PADUA; (**b**) trifecta factors according to PADUA; (**c**) trifecta status according to RENAL; (**d**) trifecta factors according to RENAL.

Overall, 193 (46%) vs. 136 (33%) vs. 89 (21%) patients were classified according to PADUA NS as low vs. moderate vs. high complexity, respectively. Conversely, 268 (64%) vs. 123 (29%) vs. 27 (7%) patients were classified according to RENAL NS as low vs. moderate vs. high complexity, respectively. Bar plots showing the distribution of trifecta achievement and determining factors according to PADUA and RENAL NS are depicted in Figure 1.

### 3.2. External Validation of PADUA and RENAL Nephrometry Scores

In the PADUA multivariable logistic regression model predicting trifecta status, only a diameter > 4 cm (odds ratio [OR]: 3.4) and the dislocation/infiltration of the urinary collecting system (OR: 2.3) reached independent predictor status (Table 2).

A bootstrapped c-index of 0.60 (0.53–0.67) determined the poor ability of the PADUA score to discriminate trifecta status.

In the RENAL multivariable logistic regression model predicting trifecta status, only a diameter > 4 cm (OR: 3.7) and the nearness to renal sinus/urinary collecting system ≤ 4 mm (OR: 6.1) reached independent predictor status. A bootstrapped c-index of 0.62 (0.55–0.69) determined the poor ability of the RENAL score to discriminate trifecta status.

### 3.3. Development of Percutaneous Thermal Ablation-Specific Nephrometry Score

In univariable logistic regression models predicting trifecta status, CSA ≥ 10 cm^2^ (OR: 3.9), nearness to renal sinus/urinary collecting system ≤ 4 mm and diameter >3 and ≤4 cm (OR: 2.7) or >4 cm (OR: 5.6) were strong predictors of trifecta status (*p* < 0.001, Supplementary Table S1).

In the multivariable logistic regression model, all the predictors maintained statistical significance (Table 3).

**Figure 2:**
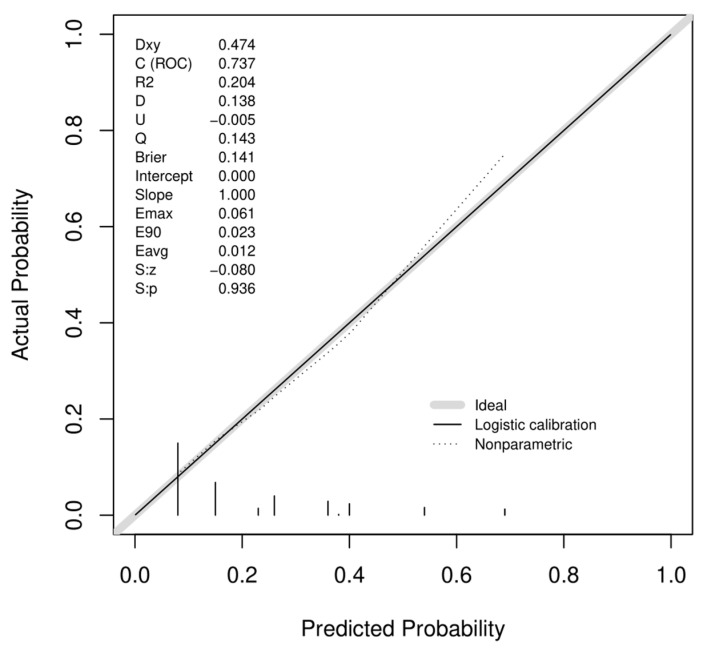
Calibration plot of observed proportions versus predicted probabilities of not achieving trifecta according to the SuNS model.

A bootstrapped c-index of 0.74 (0.67–0.79) determines the fair ability of the model to discriminate trifecta status and the calibration plot depicts a good agreement between the observed and predicted outcomes (Figure 2).

We defined three complexity classes based on logistic regression ORs associated with each score point (Supplementary Table S2). Moderate complexity (5–6 points) and high complexity (7–8 points) classes showed ORs of 3.3 and 14.3, respectively, as compared to the low complexity class (3–4 points).

**Figure 3:**
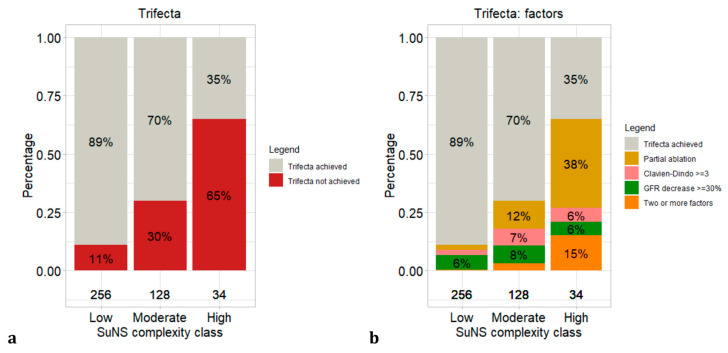
Stacked bar plots depicting trifecta not achieved and factors determining trifecta status distribution according to complexity classes: (**a**) trifecta status according to SuNS; (**b**) trifecta factors according to SuNS.

Patient distribution, distribution of trifecta achievement and determining factors according to the SuNS complexity classes are depicted in Figure 3 and Supplementary Table S3.

Specifically, the rates of no trifecta achievement were the following: 11 vs. 30 vs. 65% in low (n = 256; 61%) vs. moderate (n = 128; 31%) vs. high (n = 34; 8%) complexity, respectively. Supplementary Table S4 summarizes the percentage of patients, previously scored according to PADUA and RENAL, that were reclassified according to the SuNS score.

## 4. Discussion

To date, five NSs have been developed for the ablation-specific prediction of procedure complexity and postoperative outcomes. However, these models did not catch on in clinical practice because of a lack of formal external validation [18,19], poor ability to discriminate outcomes [15,22], difficulty in score computing or inclusion of not immediately available items [19,21,22]. In consequence, PADUA and RENAL NSs remain the established standard methods to assess complexity in nephron-sparing procedures, thanks to their immediacy and extensive validation [11,13,14,16,19,21], despite their open surgery development [7,8]. The present study aims to develop a simple, immediate, and reproducible ablation-specific NS that better predicts peri-operative outcomes compared to RENAL and PADUA NSs. Our results showed several important findings.

First, we showed the suboptimal accuracy of PADUA (c-index: 0.60) and RENAL (c-index: 0.62) NSs in predicting trifecta status. The poor discrimination ability is reflected in the distribution of trifecta status across the complexity classes. Specifically, according to PADUA complexity-class stratification, 17% vs. 18% of patients did not achieve trifecta in low vs. moderate classes, respectively. Similarly, according to RENAL complexity-class stratification, 32% vs. 37% of patients did not achieve trifecta in moderate vs. high classes, respectively. When we reproduced PADUA and RENAL multivariable models, the only items predicting trifecta status were tumour size >4 cm and nearness to renal sinus or urinary collecting system. These associations were previously detected by Maxwell et al. [16] in a similar but smaller ablation-treated cohort. These results confirm that despite PADUA and RENAL NSs providing a fair to poor peri-operative outcome discrimination ability according to many PTA cohorts [11,12,13,14,15,16,17], the majority of the items on which these scores are based do not influence outcome prediction. Based on these assumptions, testing new parameters or, at least, recoding established items related to peri-operative outcomes is needed to achieve a better prediction. However, it needs to be stated that, to the best of our knowledge, we are the first to test NS ability to predict an ablation-specific definition of trifecta. In consequence, direct comparison with other studies is not recommended.

Second, based on the aforementioned assumptions, we tested all the available outcome-related variables recorded in our dataset in a univariable fashion, recoding established NS parameters to better fit the PTA technique and selected SRM characteristics. Following the concept of complexity score, we did not include clinical patient characteristics in the univariable analysis. Of all variables tested, only size (diameter; >3 and ≤4 cm OR: 2.7, >4 cm OR: 5.6; *p* < 0.001), CSA (≥10 cm^2^ OR: 3.9; *p* < 0.001) and nearness to renal sinus or urinary collecting system (≤4 mm OR: 4.3; *p* < 0.001) resulted as strongly significant risk factors for not achieving trifecta. Specifically, we recoded PADUA and RENAL tumour size as ≤3 cm vs. >3 and ≤4 cm vs. >4 cm as suggested by previous analyses [30]. Moreover, we dichotomized the RENAL variable nearness to renal sinus or urinary collecting system in >4 mm vs. ≤4 mm, accounting for the higher precision of the PTA technique compared to PN. Third, to the best of our knowledge, CSA has never been tested in a PTA setting. This variable accurately sums up the concepts of steric tumour shape and endophicity/exophicity [23,24]. Finally, among other variables tested, the nearness to the ureter and bowel were not strong predictors of trifecta status, probably due to the standardized use of hydrodissection techniques when needed. Despite the high variety of available items tested, we were not able to validate some previously tested variables that could result as significant predictors such as, for example, the lesion enhancement pattern [16]. In consequence, future analyses should focus on these specific variables to predict trifecta status.

Third, we fitted a multivariable logistic regression model based on the three described variables: su(rface), n(earness to renal sinus or urinary collecting system), s(ize). This novel model showed good calibration and greater accuracy (c-index: 0.74) when compared to PADUA and RENAL NS. Similar to PADUA and RENAL development process, we assigned a score to each variable level based on the ORs, the total points ranging from 3 to 8. Subsequently, we generated complexity classes based on the categorical coded score ORs and trifecta achievement rates. SuNS moderate (5–6 points) and high complexity (7–8 points) classes showed an OR of 3.3 and 14.3, respectively, when low complexity (3–4 points) was the reference class. Compared to PADUA and RENAL NSs, the SuNS score discrimination ability is also reflected in a more representative distribution of patients overall (61% vs. 31% vs. 8% in low vs. moderate vs. high risk) and of patients not achieving trifecta (11% vs. 30% vs. 65% in low vs. moderate vs. high) across complexity classes. Moreover, we also observed important discrepancies in patient distribution across PADUA, RENAL and SuNS NS. For example, 40% of patients reclassified in SuNS low complexity class were classified as moderate/high class with the PADUA scoring system. Moreover, 74% of patients reclassified in high complexity class according to the SuNS score, were initially classified as low/moderate class according to RENAL. Additionally, the SuNS score can be easily adopted in clinical practice, presenting only three variables that are readily derivable from preoperative imaging. Therefore, of crucial importance in preoperative outcome predictions, is the high quality of diagnostic imaging exams, which should always consist of a CECT including urographic phases or magnetic resonance imaging. Last but not least, a sensitivity analysis assessed good accuracy for outcome prediction for both RF and MW techniques.

Taken together, we developed an immediate, simple, and reproducible ablation-specific NS (SuNS) that outperformed PADUA and RENAL NSs in predicting trifecta status.

Despite its novelty, our study is not devoid of limitations. First, the current data are retrospective and influenced by inherent selection bias. Second, this study represents the practice of a high-volume oncological referral centre, and our results might hardly be generalizable. Third, the study was a single-centre evaluation, potentially creating bias in techniques or outcomes. Fourth, due to missing data, we were unable to perform a formal comparison with other NSs previously reported [9,10,18,19,21,22]. Fourth, despite bootstrap resampling, the lack of external validation currently limits predictions concerning the possible impact of our NS in clinical practice. Fifth, our cohort did not involve patients treated with cryoablation and further validation in this setting is needed to extend the SuNS score application. Last, we lack other important PTA outcomes, such as ablation time and local recurrences, which should, therefore, be tested in future analyses.

## 5. Conclusions

We developed an immediate, simple, and reproducible ablation-specific NS (SuNS) that outperformed PADUA and RENAL NSs in predicting peri-operative outcomes. External validation is required before daily practice implementation.

## Figures and Tables

**Table 1:** Descriptive characteristics of 418 patients diagnosed with small renal masses between 2008 and 2021 and treated with ablation. Stratification was made according to trifecta status (trifecta achieved vs. trifecta not achieved). Data are shown as medians for continuous variables or as counts and percentages (%) for categorical variables.

Small Renal Masses Treated with Ablation	Overall n = 418	Trifecta Achieved n = 329 (79%)	Trifecta Not Achieved n = 89 (21%)	*p*-Value
**Age at diagnosis (years)**				**<0.001**
Median (IQR)	67 (59–76)	67 (59–74)	73 (63–80)	
Sex				**0.04**
Male	277 (66%)	226 (69%)	51 (57%)	
**Age-adjusted Charlson Comorbidity Index**				**0.01**
Median (IQR)	5 (4–6)	5 (4–6)	6 (4–7)	
**Body mass index**				0.9
Median (IQR)	26.0 (24.0–29.0)	26.0 (23.9–29.0)	26.3 (24.0–28.0)	
**Side**				0.6
Left	180 (43%)	145 (44%)	35 (39%)	
Right	210 (50%)	161 (49%)	49 (55%)	
Bilateral	28 (7%)	23 (7%)	5 (6%)	
**Face**				0.3
Posterior	215 (51%)	165 (50%)	50 (56%)	
Anterior	203 (49%)	164 (50%)	39 (44%)	
**Renal rim**				0.1
Lateral	255 (61%)	207 (63%)	48 (54%)	
Medial	163 (39%)	122 (37%)	41 (46%)	
**Polar location**				0.3
Upper	115 (28%)	85 (26%)	30 (34%)	
Middle	169 (40%)	134 (41%)	35 (39%)	
Lower	134 (32%)	110 (33%)	24 (27%)	
**cT stage**				**<0.001**
T1a	370 (89%)	304 (92%)	66 (74%)	
T1b	48 (11%)	25 (8%)	23 (26%)	
**Size (diameter)**				**<0.001**
≤3 cm	289 (69%)	248 (75%)	41 (46%)	
>3 ≤ 4 cm	81 (19%)	56 (17%)	25 (28%)	
>4 cm	48 (11%)	25 (8%)	23 (26%)	
**Contact surface area**				**<0.001**
<10 cm^2^	218 (52%)	194 (59%)	24 (27%)	
≥10 cm^2^	200 (48%)	135 (41%)	65 (73%)	
**Nearness to renal sinus or collecting system**				**<0.001**
>4 mm	331 (79%)	280 (85%)	51 (57%)	
≤4 mm	87 (21%)	49 (15%)	38 (43%)	
**Nearness to ureter (mm)**				**0.004**
Median (IQR)	53 (40–66)	55 (42–66)	48 (37–58)	
**Nearness to bowel (mm)**				0.4
Median (IQR)	49 (32–69)	48 (31, 69)	50 (34, 66)	
**Skin-to-tumour distance**				0.3
≤10 cm	299 (72%)	239 (73%)	60 (67%)	
>10 cm	119 (28%)	90 (27%)	29 (33%)	
**Histology**				0.2
Clear cell	240 (62%)	190 (62%)	50 (65%)	
Non-clear cell	97 (25%)	83 (27%)	14 (18%)	
Benign	48 (12%)	35 (11%)	13 (17%)	
**Technique**				**0.01**
Microwave	268 (64%)	221 (67%)	47 (53%)	
Radiofrequency	150 (36%)	108 (33%)	42 (47%)	
**PADUA complexity class**				**<0.001**
Low	193 (46%)	161 (49%)	32 (36%)	
Moderate	136 (33%)	111 (34%)	25 (28%)	
High	89 (21%)	57 (17%)	32 (36%)	
**RENAL complexity class**				**<0.001**
Low	268 (64%)	228 (69%)	40 (45%)	
Moderate	123 (29%)	84 (26%)	39 (44%)	
High	27 (7%)	17 (5%)	10 (11%)	
**Ablation**				**<0.001**
Complete	376 (90%)	329 (100%)	47 (53%)	
Partial	42 (10%)	0 (0%)	42 (47%)	
**Clavien–Dindo class**				**<0.001**
<3	394 (94%)	329 (100%)	65 (73%)	
≥3	24 (6%)	0 (0%)	24 (27%)	
**Glomerular filtration-rate decrease**				**<0.001**
<30%	385 (92%)	329 (100%)	56 (63%)	
≥30%	33 (8%)	0 (0%)	33 (37%)	
**Reason for trifecta not achieved**				**<0.001**
Trifecta achieved	329 (79%)	329 (100%)	0 (0%)	
Partial ablation	34 (8%)	0 (0%)	34 (38%)	
Glomerular filtration-rate decrease ≥ 30%	28 (7%)	0 (0%)	28 (31%)	
Clavien–Dindo class ≥ 3	17 (4%)	0 (0%)	17 (19%)	
Two or more factors	10 (2%)	0 (0%)	10 (11%)	

Bold values indicate statistical significance *p* < 0.05. IQR: interquartile range.

**Table 2:** Separate multivariable logistic regression models for PADUA and RENAL predicting trifecta status (trifecta achieved vs. trifecta not achieved) in patients diagnosed with small renal masses between 2008 and 2021 and treated with ablation. Bootstrapped c-index represents the discrimination ability of the score generated by the models.

PADUA Derived Score c-Index: 0.60 (0.53–0.67)				RENAL Derived Score c-Index: 0.62 (0.55–0.69)			
Model Items	Odds Ratio	95% CI	*p*-Value	Model Items	Odds Ratio	95% CI	*p*-Value
**Polar location**				**Polar location**			
Upper/Lower	Ref			Entirely above or below the polar lines	Ref		
				Crosses a polar line	1.0	(0.5–1.8)	0.9
Middle	0.8	(0.5–1.4)	0.5	>50% crosses a polar line or crosses the axial renal midline or entirely between the polar lines	0.6	(0.3–1.4)	0.3
**Exophytic rate**				**Exophytic rate**			
<50%	Ref			<50%	Ref		
≥50%	1.1	(0.6–2.1)	0.7	≥50%	1.2	(0.6–2.3)	0.5
Endophytic	0.9	(0.4–1.9)	0.8	Endophytic	0.7	(0.3–1.6)	0.4
**Size (diameter)**				**Size (diameter)**			
≤4 cm	Ref			≤4 cm	Ref		
>4 cm	3.4	(1.7–6.8)	**<0.001**	>4 cm	3.7	(1.8–7.5)	**<0.001**
**Renal sinus**				**Nearness to renal sinus or collecting system**			
Not involved	Ref			≥7 mm	Ref		
				4–7 mm	1.3	(0.6–2.5)	0.5
Involved	1.0	(0.5–2.1)	0.9	≤4 mm	6.1	(3.0–12.5)	**<0.001**
**Urinary collecting system**							
Not involved	Ref						
Dislocated/infiltrated	2.3	(1.2–4.5)	**0.01**				
**Renal rim**							
Lateral	Ref						
Medial	1.3	(0.8–2.2)	0.3				

Bold values indicate statistical significance *p* < 0.05. CI: confidence interval.

**Table 3:** Multivariable logistic regression models predicting trifecta status (trifecta achieved vs. trifecta not achieved) in patients diagnosed with small renal masses between 2008 and 2021 and treated with ablation. Bootstrapped c-index represents the discrimination ability of the score generated by the models.

SuNS Score Derived Score c-Index: 0.74 (0.67–0.79)	Odds Ratio	95% CI	*p*-Value	Points Assigned
**Contact surface area**				
<10 cm^2^	Ref			1
≥10 cm^2^	1.9	(1.04–3.5)	**0.04**	2
**Nearness to renal sinus or collecting system**				
>4 mm	Ref			1
≤4 mm	3.3	(1.9–5.8)	**<0.001**	3
**Size (diameter)**				
≤3 cm	Ref			1
>3 ≤ 4 cm	2.0	(1.1–3.8)	**0.03**	2
>4 cm	3.8	(1.8–8.0)	**<0.001**	3

Bold values indicate statistical significance *p* < 0.05. SuNS: surface, nearness to renal sinus or collecting system, size; CI: confidence interval.

## Data Availability

The data sets generated during and/or analyzed during the current study are available from the corresponding author upon reasonable request.

## Supplementary Materials

The following supporting information can be downloaded at: https://www.mdpi.com/article/10.3390/diagnostics13182955/s1, Supplementary Figure S1: Stacked bar plots depicting trifecta not achieved according to complexity classes: (a) trifecta status according to SuNS in microwave-treated patients; (b) trifecta status according to SuNS in radiofrequency treated patients; Supplementary Table S1: Separate univariable logistic regression models predicting trifecta status (trifecta achieved vs. trifecta not achieved) in patients diagnosed with small renal masses between 2008 and 2021 and treated with ablation; Supplementary Table S2: Rates of trifecta achieved and univariable logistic regression predicting trifecta status (trifecta achieved vs. trifecta not achieved) according to SuNS score and derived complexity classes; Supplementary Table S3: Descriptive characteristics of 418 patients diagnosed with small renal masses between 2008 and 2021 and treated with ablation. Stratification is made according to SuNS score complexity class (low vs. moderate vs. high); Supplementary Table S4: Comparison of patient distribution according to complexity class between PADUA, RENAL and SuNS scores.
