# Supplementary material for: Predicting Peri-Operative Outcomes in Patients Treated with Percutaneous Thermal Ablation for Small Renal Masses: The SuNS Nephrometry Score
Source: Diagnostics (Basel). 2023 Sep 15;13(18):2955. doi: 10.3390/diagnostics13182955 (PMC10528095; doi:10.3390/diagnostics13182955)
Supplement: Supplementary file 1 [file diagnostics-13-02955-s001.zip › Supplementary table S1.pdf]

Supplementary Table S1. Separate univariable logistic regression models predicting trifecta status (trifecta achieved vs trifecta not achieved) in patients diagnosed with small renal masses between 2008 and 2021 and treated with ablation. C-index represents the discrimination ability of the variable.

Variables tested	Odds ratio	95% CI	p-value	C-index
**Side**				0.53
Left	Ref			
Right	1.3	(0.8-2.1)	0.3	
Bilateral	0.9	(0.3-2.4)	0.8	
**Face**				0.53
Posterior	Ref			
Anterior	0.8	(0.5-1.2)	0.3	
**Renal rim**				0.54
Lateral	Ref			
Medial	1.4	(0.9-2.3)	0.1	
**Polar location**				0.55
Upper	Ref			
Middle	0.7	(0.4-1.3)	0.3	
Lower	0.6	(0.3-1.1)	0.1	
**Size (diameter)**				0.66
≤3 cm	Ref			
>3 ≤4 cm	2.7	(1.5-4.8)	**<0.001**	
>4 cm	5.6	(2.9-10.8)	**<0.001**	
**Contact surface area**				0.66
<10 cm^2	Ref			
≥10 cm^2	3.9	(2.3-6.6)	**<0.001**	
**Nearness to renal sinus or collecting system**				0.64
>4 mm	Ref			
≤4 mm	4.3	(2.5-7.2)	**<0.001**	
**Nearness to ureter**				0.58
>6 cm	Ref			
4.5-6 cm	1.4	(0.7-2.9)	0.3	
<4.5 cm	2.2	(1.2-4.3)	**0.02**	
**Nearness to bowel**				0.51
>6 cm	Ref			
4-6 cm	1.03	(0.6-1.9)	0.9	
<4 cm	0.9	(0.5-1.6)	0.7	
**Skin-to-tumour distance**				0.53
≤10 cm	Ref			
>10 cm	1.3	(0.8-2.1)	0.3	
**Histology**				0.56
Clear cell	Ref			
Non-clear cell	0.6	(0.3-1.2)	0.2	
Benign	1.4	(0.7-2.8)	0.3	

Bold values indicate statistical significance p<0.05.

CI: confidence interval.
