# Supplementary material for: Predicting Peri-Operative Outcomes in Patients Treated with Percutaneous Thermal Ablation for Small Renal Masses: The SuNS Nephrometry Score
Source: Diagnostics (Basel). 2023 Sep 15;13(18):2955. doi: 10.3390/diagnostics13182955 (PMC10528095; doi:10.3390/diagnostics13182955)
Supplement: Supplementary file 1 [file diagnostics-13-02955-s001.zip › Supplementary table S2.pdf]

Supplementary Table S2 Rates of trifecta achieved and univariable logistic regression predicting trifecta status (trifecta achieved vs trifecta not achieved) according to SuNS score and derived complexity classes.

SuNS score	Rate of trifecta achieved	Odds ratio	p-value	Complexity classes	Odds ratio	p-value
**3**	91%	Ref		**Low**	Ref	
**4**	84%	1.9	0.1			
**5**	77%	3.1	**0.005**	**Moderate**	3.3	**<0.001**
**6**	64%	5.6	**<0.001**			
**7**	47%	11.1	**<0.001**	**High**	14.3	**<0.001**
**8**	20%	40.0	**<0.001**			

Bold values indicate statistical significance $p<0.05$.

SuNS: Su(rface), N(earness to renal sinus or collecting system), S(ize); CI: confidence interval.
