# Supplementary material for: Predicting Peri-Operative Outcomes in Patients Treated with Percutaneous Thermal Ablation for Small Renal Masses: The SuNS Nephrometry Score
Source: Diagnostics (Basel). 2023 Sep 15;13(18):2955. doi: 10.3390/diagnostics13182955 (PMC10528095; doi:10.3390/diagnostics13182955)
Supplement: Supplementary file 1 [file diagnostics-13-02955-s001.zip › Supplementary table S3.pdf]

Supplementary Table S3. Descriptive characteristics of 418 patients diagnosed with small renal masses between 2008 and 2021 and treated with ablation. Stratification is made according to SuNS score complexity class (low vs moderate vs high). Data are shown as medians for continuous variables or as counts and percentages (%) for categorical variables.

Renal cell carcinoma treated with ablation	Overall n = 418	Low n = 256 (61%)	Moderate n = 128 (31%)	High n = 34 (8%)	p-value
**Age at diagnosis (years)**	67 (59-76)	67 (59-74)	69 (62-78)	70 (63-80)	**0.04**
Median (IQR)					
**Sex**					0.12
Male	277 (66%)	179 (70%)	76 (59%)	22 (65%)	
**Age-adjusted Charlson Comorbidity Index**	5.00 (4.00-6.00)	5.00 (4.00-6.00)	5.00 (4.00-6.25)	5.50 (4.00-7.75)	0.2
Median (IQR)					
**Body mass index**	26.0 (24.0-29.0)	25.6 (23.6-28.0)	26.9 (24.4-29.4)	27.3 (25.0-28.0)	**0.01**
Median (IQR)					
**Side**					0.5
Left	180 (43%)	104 (41%)	60 (47%)	16 (47%)	
Right	210 (50%)	137 (54%)	57 (45%)	16 (47%)	
Bilateral	28 (7%)	15 (5%)	11 (8%)	2 (6%)	
**Face**					0.3
Posterior	215 (51%)	133 (52%)	69 (54%)	13 (38%)	
Anterior	203 (49%)	123 (48%)	59 (46%)	21 (62%)	
**Renal rim**					**0.008**
Lateral	255 (61%)	171 (67%)	68 (53%)	16 (47%)	
Medial	163 (39%)	85 (33%)	60 (47%)	18 (53%)	
**Polar location**					0.05
Upper	115 (28%)	71 (28%)	34 (27%)	10 (29%)	
Middle	169 (40%)	94 (37%)	55 (43%)	20 (59%)	
Lower	134 (32%)	91 (36%)	39 (30%)	4 (12%)	
**cT stage**					**<0.001**
T1a	370 (89%)	256 (100%)	95 (74%)	19 (56%)	
T1b	48 (11%)	0 (0%)	33 (26%)	15 (44%)	
**Size (diameter)**					**<0.001**
≤3 cm	289 (69%)	238 (93%)	51 (40%)	0 (0%)	
>3 ≤4 cm	81 (19%)	18 (7%)	44 (34%)	19 (56%)	
>4 cm	48 (11%)	0 (0%)	33 (26%)	15 (44%)	
**Contact surface area**					**<0.001**
<10 cm^2	218 (52%)	194 (76%)	24 (19%)	0 (0%)	
≥10 cm^2	200 (48%)	62 (24%)	104 (81%)	34 (100%)	
**Nearness to renal sinus or collecting system**					**<0.001**
>4 mm	331 (79%)	256 (100%)	75 (59%)	0 (0%)	
≤4 mm	87 (21%)	0 (0%)	53 (41%)	34 (100%)	
**Nearness to ureter (mm)**	53 (40-66)	56 (43-67)	51 (38-65)	43 (35-58)	**0.009**
Median (IQR)					
**Nearness to bowel (mm)**	49 (32-69)	46 (30, 67)	53 (34, 73)	49 (35-63)	0.3
Median (IQR)					
**Skin-to-tumour distance**					0.2
≤10 cm	299 (72%)	191 (75%)	86 (67%)	22 (65%)	
>10 cm	119 (28%)	65 (25%)	42 (33%)	12 (35%)	
**Histology**					0.06
Clear cell	240 (62%)	135 (57%)	80 (68%)	25 (80%)	
Non-clear cell	97 (25%)	69 (29%)	25 (21%)	3 (10%)	
Benign	48 (12%)	32 (14%)	13 (11%)	3 (10%)	
**Technique**					**0.04**
Microwave	268 (64%)	175 (68%)	76 (59%)	17 (50%)	
Radiofrequency	150 (36%)	81 (32%)	52 (41%)	17 (50%)	
**Ablation**					**<0.001**
Complete	376 (90%)	249 (97%)	111 (87%)	16 (47%)	
Partial	42 (10%)	7 (3%)	17 (13%)	18 (53%)	
**Clavien-Dindo class**					**0.002**
<3	394 (94%)	249 (97%)	116 (91%)	29 (85%)	
≥3	24 (6%)	7 (3%)	12 (9%)	5 (15%)	
**Glomerular filtration rate decrease**					0.2
<30%	385 (92%)	240 (94%)	115 (90%)	30 (88%)	
≥30%	33 (8%)	16 (6%)	13 (10%)	4 (12%)	
**Reason for trifecta not achieved**					**<0.001**
Trifecta achieved	329 (79%)	227 (89%)	90 (70%)	12 (35%)	
Partial ablation	34 (8%)	6 (2%)	15 (12%)	13 (38%)	
Glomerular filtration rate decrease ≥30%	28 (7%)	6 (2%)	9 (7%)	2 (6%)	
Clavien-Dindo class ≥3	17 (4%)	16 (6%)	10 (8%)	2 (6%)	
Two or more factors	10 (2%)	1 (1%)	4 (3%)	5 (15%)	

Bold values indicate statistical significance p<0.05.

SuNS: Su(rface), N(earness to renal sinus or collecting system), S(ize); IQR: interquartile range.
