# Supplementary material for: Predicting Peri-Operative Outcomes in Patients Treated with Percutaneous Thermal Ablation for Small Renal Masses: The SuNS Nephrometry Score
Source: Diagnostics (Basel). 2023 Sep 15;13(18):2955. doi: 10.3390/diagnostics13182955 (PMC10528095; doi:10.3390/diagnostics13182955)
Supplement: Supplementary file 1 [file diagnostics-13-02955-s001.zip › Supplementary table S4.pdf]

Supplementary Table S4. Comparison of patients distribution according to complexity class between PADUA, RENAL and SuNS scores.

**Complexity classes**	**SuNS low complexity n = 256 (61%)**		**SuNS moderate complexity n = 128 (31%)**		**SuNS high complexity n = 34 (8%)**	
	**PADUA**	**RENAL**	**PADUA**	**RENAL**	**PADUA**	**RENAL**
**Low**	155 (60%)	214 (84%)	38 (30%)	52 (41%)	0 (0%)	2 (6%)
**Moderate**	84 (33%)	42 (16%)	38 (30%)	58 (45%)	14 (41%)	23 (68%)
**High**	17 (7%)	0 (0%)	52 (40%)	18 (14%)	20 (59%)	9 (26%)

SuNS: Su(rface), N(earness to renal sinus or collecting system), S(ize)
