# Supplementary figures and images for: Predicting Peri-Operative Outcomes in Patients Treated with Percutaneous Thermal Ablation for Small Renal Masses: The SuNS Nephrometry Score
Source: Diagnostics (Basel). 2023 Sep 15;13(18):2955. doi: 10.3390/diagnostics13182955 (PMC10528095; doi:10.3390/diagnostics13182955)

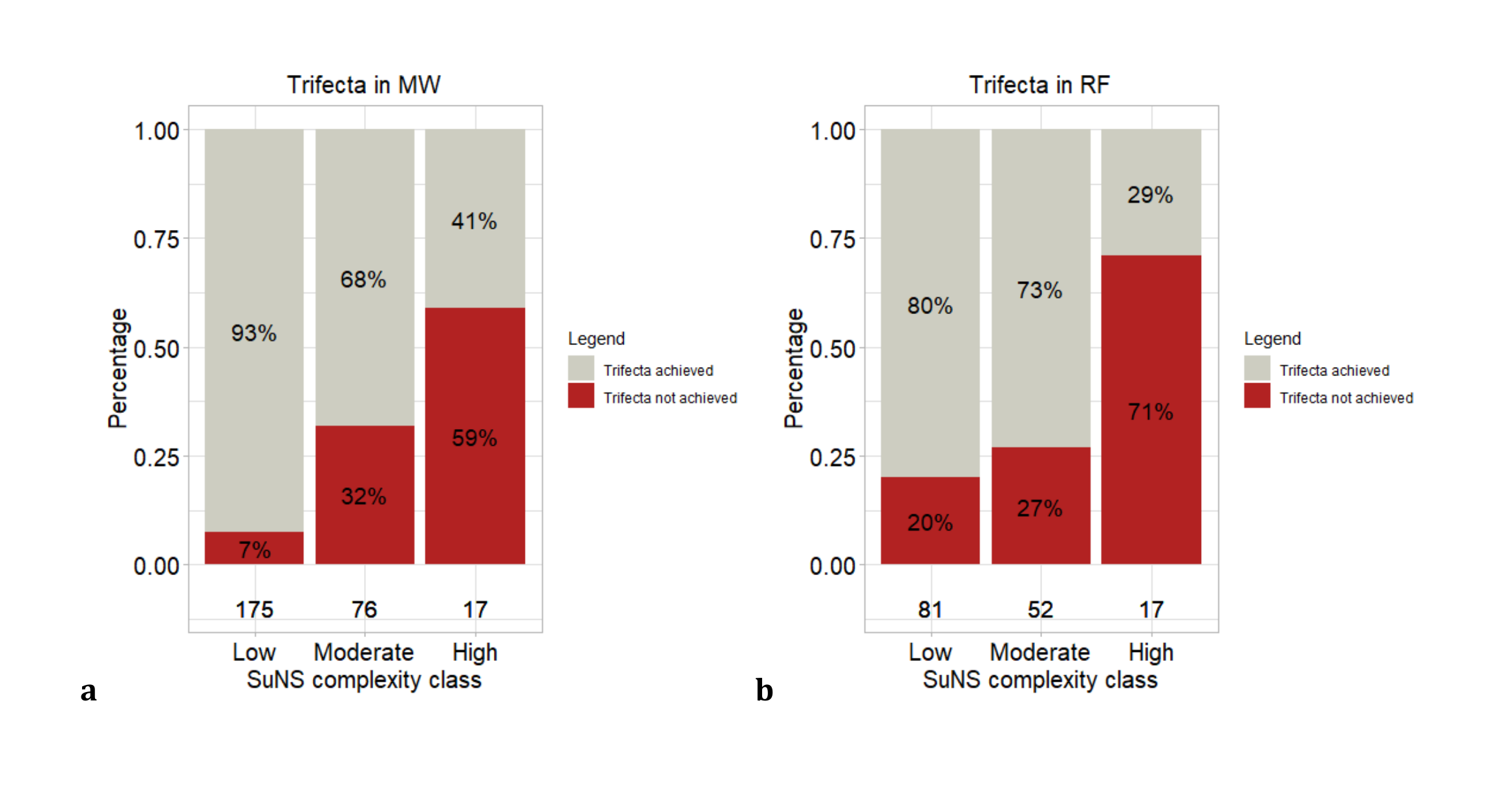

Supplement: Supplementary file 1 [file diagnostics-13-02955-s001.zip › Supplementary figure 1.tiff]
